# Synaptojanin1 Modifies Endolysosomal Parameters in Cultured Ventral Midbrain Neurons

**DOI:** 10.1523/ENEURO.0426-22.2023

**Published:** 2023-05-04

**Authors:** Xinyu Zhu, Sanjana Surya Prakash, Geoffrey McAuliffe, Ping-Yue Pan

**Affiliations:** Department of Neuroscience and Cell Biology, Robert Wood Johnson Medical School, Rutgers University, Piscataway, NJ 08854

**Keywords:** α-synuclein, autophagy, lysosome, synaptojanin1

## Abstract

The accumulation of α-synuclein (α-syn)-enriched protein aggregates is thought to arise from dysfunction in degradation systems within the brain. Recently, missense mutations of *SYNJ1* encoding the SAC1 and 5’-phosphatase domains have been found in families with hereditary early-onset Parkinsonism. Previous studies showed that *Synj1* haploinsufficiency (*Synj1*+/−) leads to accumulation of the autophagy substrate p62 and pathologic α-syn proteins in the midbrain (MB) and striatum of aged mice. In this study, we aim to investigate the neuronal degradation pathway using the *Synj1*+/− MB culture from mouse pups of mixed sex as a model. Our data show that GFP-LC3 puncta formation and cumulative mKeima puncta formation are unaltered at baseline in *Synj1*+/− MB neurons. However, GFP-LAMP1 puncta is reduced with a similar decrease in endogenous proteins, including lysosomal-associated membrane protein (LAMP)1, LAMP2, and LAMP2A. The LAMP1 vesicles are hyperacidified with enhanced enzymatic activity in *Synj1*+/− MB neurons. Using a combination of light and electron microscopy (EM), we show that endolysosomal changes are primarily associated with a lack of SAC1 activity. Consistently, expressing the SYNJ1 R258Q mutant in N2a cells reduces the lysosome number. Interestingly, the endolysosomal defects in *Synj1*+/− neurons does not impact the clearance of exogenously expressed wild-type (WT) α-syn; however, the clearance of α-syn A53T was impaired in the axons of *Synj1*+/− MB neurons. Taken together, our results suggest axonal vulnerability to endolysosomal defects in Synj1-deficient MB neurons.

## Significance Statement

The study by Zhu et al. discovered a previously uncharacterized role of Synj1 in regulating endolysosomal number, protein, and acidity in ventral midbrain neurons. These alterations are associated with a specific impairment in the clearance of α-synuclein (α-syn) A53T, but not wild-type (WT) α-syn in axons, suggesting an essential role of Synj1 in axonal degradative capacity under pathologic stress. This work in cultured mammalian neurons complements recent research efforts in *Drosophila*, *Caenorhabditis elegans*, and zebrafish and provides a novel insight for the role Synj1 in neuronal degradation.

## Introduction

Parkinson’s disease (PD) is characterized by progressive loss of substantia nigra pars compacta (SNpc) dopamine (DA) neurons and the accumulation of Lewy bodies enriched in pathologic forms of α-synuclein (α-syn; Surmeier et al., 2017; Oliveira et al., 2021; Henderson et al., 2022). While the exact cause of SNpc neuron loss remains elusive, bioinformatics analyses on familial inherited PD and sporadic PD have identified PD genes and risk loci that congregate in the autolysosomal pathway (Robak et al., 2017; Vázquez-Vélez and Zoghbi, 2021; Fleming et al., 2022; Udayar et al., 2022). Macroautophagy (hereafter referred to as “autophagy” in this study) is a “self-eating” process that degrades a wide range of cytosolic components including misfolded proteins and damaged organelles to maintain cellular homeostasis (Klionsky, 2007; Nixon, 2013). This process involves the formation of mature autophagosomes followed by fusion with endolysosomes for degradation of cytosolic contents. Chaperone-mediated autophagy (CMA) is mediated by lysosomal-associated membrane protein (LAMP)2A-assisted protein translocation (Kon and Cuervo, 2010; Kaushik and Cuervo, 2018). It is less common but highly important for degradation of α-syn (Cuervo et al., 2004). Whether and how each individual PD gene is implicated in the autophagosome/lysosome dysfunction during pathogenesis remains to be elucidated.

Recently, missense mutations including R258Q, R459P and R839C in PARK20/*SYNJ1* have been discovered in families with early onset parkinsonism (Krebs et al., 2013; Quadri et al., 2013; Kirola et al., 2016; Taghavi et al., 2018). Reduced *SYNJ1* transcripts were also reported in a subset of sporadic PD brains (Pan et al., 2020). *SYNJ1* encodes synaptojanin1/Synj1, a phosphoinositol phosphatase with two enzymatic domains: a 5’-phosphotase domain that specifically hydrolyzes the 5’-phosphate from the inositol ring of PI(4,5)P_2_ (Guo et al., 1999; Pirruccello and De Camilli, 2012) and a SAC1-like domain that dephosphorylates PI3P, PI4P, and PI(3,5)P_2_ into PI (Zhong et al., 2012). Interestingly, Synj1 was initially characterized to cooperate with endophilin to regulate synaptic vesicle recycling through regulating plasma membrane PI(4,5)P_2_ levels and nascent clathrin coated vesicles (McPherson et al., 1996; Cremona et al., 1999; Verstreken et al., 2002, 2003). Since the identification of *SYNJ1* mutations in PD, new investigations have emerged that examined the roles of Synj1 in the autolysosomal pathway. Recent studies in *Drosophila* and *Caenorhabditis elegans* showed that the SAC1 domain is required for autophagosome formation. The R258Q mutation, which abolishes SAC1 enzymatic activity, delays autophagosome maturation because of an accumulation of PI(3)P and PI(3,5)P_2_ and/or disrupted Atg9 activity (Vanhauwaert et al., 2017; Yang et al., 2022). In cultured mouse cortical astrocytes, however, an increase in autophagosome formation was observed in cells from the Synj1-deficient background (Pan et al., 2021). Autophagy impairment was found in the later stages of autolysosomal degradation, and both phosphatase domains were involved in stress-induced clearance of p62 (Pan et al., 2021). Consistently, in aged *Synj1* haploinsufficient (*Synj1*+/−) mice an accumulation of the autophagy substrate p62 was observed in all brain regions including DA neurons in the midbrain (Pan et al., 2020). Additionally, in the cone photoreceptors of zebrafish retina, Synj1 deletion led to mislocalization and abnormal accumulation of late endosomes and autophagosomes (George et al., 2014, 2016). While these recent studies present evidence for a novel role of Synj1 in regulating the autophagic and autolysosomal functions, results obtained from different model systems are somewhat conflicting.

*SYNJ1* downregulation was identified in a subset of sporadic PD patients (Pan et al., 2020), and we therefore decided to investigate Synj1 heterozygous (*Synj1*+/−) deficiency, which represents a more prevalent genetic condition than homozygous point mutations found in few families so far. In this study, we used multiple optical tools to analyze autophagosome and endolysosome structures in cultured *Synj1*+/− midbrain neurons. We found that Synj1 haploinsufficiency did not affect the basal level LC3-mediated autophagy flux in the soma of ventral MB neurons. Robust reduction was observed for the GFP-LAMP1 puncta number, endogenous LAMP1, LAMP2 and LAMP2a proteins, accompanied by endolysosomal hyperacidification. Further, we found the endolysosomal defect is associated with a reduction in SAC1 activity rather than 5’-phosphase activity. More interestingly, the degradation of α-syn A53T, but not WT α-syn, was significantly impaired in the axons of *Synj1*+/− neurons. Taken together, our results indicate specific vulnerability in axonal degradation in response to Synj1 deficiency associated with endolysosomal dysfunction.

## Materials and Methods

### Plasmids, antibodies, and reagents

The following plasmids, pEGFP-C1-GFP-Synj1 WT, pEGFP-C1-GFP-Synj1 R258Q, pEGFP-C1-GFP-Synj1 R839C, pEGFP-C1-GFP-Synj1 D769A, pEGFP-C1-GFP-LC3 were previously reported (Pan et al., 2020, 2021). mKeima was gifted by Atsushi Miyawaki (Osaka University). The plasmid expressing LAMP1-GFP is a gift from Prof. Tao Xu (Institute of biophysics, Chinese Academy of Sciences). The FIRE-pHLy plasmid was purchased from Addgene (catalog #170775). Mouse WT or A53T α-syn cDNAs (gifted by Eliezer Masliah, National Institute on Aging) was cloned into pmEos3.2-N1 vector (from Addgene, catalog #54525) to generate the α-syn-mEos3.2/α-syn A53T-mEos3.2 plasmid.

The GlutaMAX Supplement (35050061), the Neurobasal-A Medium (12349015), the B-27 Supplement (17504044), the basal medium Eagle (BME; 21010046), the DMEM (11965-092), the penicillin-streptomycin (15140122), the Lipofectamine 2000 Reagent (11668019), the Lipofectamine 3000 Transfection Reagent (L3000150), the DQ Red BSA (D12051), the LAMP-2A Polyclonal Antibody (51-2200, 1:100 for IF), the LAMP2 Polyclonal Antibody (PA1-655, 1:200 for IF), the LAMP-1 Monoclonal Antibody (14-1071-85, 1:500 for IF), the MAP2 Monoclonal Antibody (MAP2 MA5-12826, 1:1000 for IF), and all of the Alexa Fluor dye-conjugated secondary antibodies were purchased from Thermo Scientific. The Magic Red Cathepsin-B Assay kit (MR; 937) was purchased from Immunochemistry. The mouse Cathepsin B Antibody (AF965; 1:200 for IF) was purchased from R&D Systems, Inc. The anti-MAP2 Polyclonal Chicken Antibody (188006, 1:1000 for IF) and the anti-MAP2 Polyclonal Guinea Pig Antibody (188004, 1:1000 for IF) were purchased from Synaptic Systems. The anti-Tyrosine Hydroxylase Antibody (AB9702, 1:1000 for IF) was purchased from Millipore Sigma. The papain (LK003176) and the Earle’s balanced salt solution (EBSS; LK003188) were purchased from Worthington Biochemical Corporation. The fetal bovine serum (FBS; S11510H) was purchased from Novus Biologicals.

### Cell sorting and EM analysis

The N2a cells transfected with GFP-Synj1 WT or GFP-Synj1 R258Q on day 1 were sorted to collect GFP expressing cells by an fluorescence-activated cell sorting (FACS) equipment on day 2. Then, on day 4, the cells were fixed in the culture dish by replacing the culture medium with 2.5% glutaraldehyde in 0.1 m sodium cacodylate buffer containing 2.5 mm Ca^2+^ at room temperature (RT). After fixation for 60–90 min, the cells were rinsed three times with the same buffer, and then postfixed for 60 min with 1% osmium in the rinsing buffer containing 1% potassium ferricyanide. The cells were rinsed again with rinsing buffer and stained with 1% uranyl acetate at 4°C for 30 min. Dehydration with graded ethanol solutions was followed by infiltration and embedding with PolyBed812. After polymerization portions of the embedment were cut out and re-embedded in Polybed812. So, the cells could be sectioned perpendicular to the dish surface. Thin sections were viewed in a Philips CM12 electron microscope (EM) to capture images.

The diameter of lysosome was measured by drawing a line manually with the tool Straight Line in Fiji. To measure the density of lysosome, lysosome number was counted and divided by the entire cytoplasmic area defined in ImageJ.

### Animal

The *Synj1+/−* mice were originally from the Pietro De Camilli laboratory at Yale University (Cremona et al., 1999). They were housed and handled in accordance with the National Institutes of Health guidelines approved by the Institutional Animal Care and Use Committee. Approved IACUC protocol: PROTO201800183. C57bl/6 mice (Jax catalog #000664) and *Synj1*+/− mice were used as breeders to generate *Synj1+/+* and *Synj1*+/− pups identified by genotyping. Both male and female pups were used for cell culture.

### Immunofluorescence (IF)

The immunostaining protocol was previously described (Zhu et al., 2018; Pan et al., 2020) with minor modifications. Briefly, the cells were fixed with 4% PFA for 15 min at room temperature (RT), permeabilized with 0.5% saponin for 15 min at RT, blocking with 5% BSA for 1 h at RT, incubated with primary antibody overnight and incubated with fluorescent dye conjugated secondary antibody for 1 h at RT. After complete washing with PBS, the slides were mounted with Clear-Mount (EMS, 17985-12).

### MR and BSA-DQ analysis in MB culture

For MR staining, the MB culture was incubated with MR at 1:100 dilution in culture medium for 1 h, then the culture was subjected to live cell confocal imaging. For BSA-DQ staining, the MB culture was incubated with BSA-DQ (40 μg/ml diluted in culture medium) for 6 h, then the culture was subjected to live cell imaging. Glial cells expressing GFP were used as reference to record the stage locations of the imaged cells, whose neuronal identification was confirmed by MAP2 immunoreactivity during *post hoc* analysis guided by the stage location.

### Midbrain culture preparation and transfection

The preparation of mouse ventral midbrain culture was described before (Pan et al., 2017, 2020). Briefly, after genotyping, the ventral midbrain tissue was dissected from postnatal day 0–1 littermates, and it was digested in a papain solution (21 U/ml, prepared with EBSS supplemented with 5 mm kynurenic acid) by stirring with a magnetic stir bar at 33–37°C for 12 min under humidified oxygenation. After spinning and discarding the digestion buffer, the collected tissue chunk was triturated by using a 1 ml pipette tip in Neuro A complete medium (0.6× Neurobasal-A Medium, 0.3× BME, 1× GlutaMAX Supplement, 1× B-27 Supplement, and 10% FBS). The homogenized tissue was collected by spinning and the supernatant containing debris was discarded. The pellet was resuspended with Neuro A complete medium and subjected for spinning again. Then, the supernatant was discarded, and the pellet was resuspended with Neuro A complete medium. After counting, 25,000 cells were seeded into a 6 × 8 mm cylinder. On day 3, medium was changed with fresh Neuro A complete medium with Ara-C (0.2 μm) to inhibit glia growth. Transfection was performed on days 3–5 using calcium phosphate solution, or on days 6–8 using Lipofectamine 2000 reagent or Lipofectamine 3000 Reagent. In either case, 0.7 μg DNA was transfected for each 6 × 8 mm area of culture.

### N2a cell culture and transfection

N2a cells are cultured in 10% FBS contained DMEM medium supplemented with 10 U/ml penicillin-streptomycin. Passage procedure follows the general and standard culture protocol with digestion using 0.05% Trypsin-EDTA solution. Lipofectamine 2000 or Lipofectamine 3000 was used for plasmid transfection in N2a cells.

### Imaging and data analysis

All fluorescent images were captured using a Nikon CREST spinning disk confocal microscope with a 100×, 60×, or 40× oil objective. And all the image analysis was performed by ImageJ/Fiji. For analyzing the immunofluorescence of LAMP1, LAMP2A, LAMP2, and Magic Red in neuron soma of glia-neuron culture, the floated neurons were selected for imaging. To further avoid the immunofluorescence contamination from glia, the upper confocal planes, no longer including fluorescent signal of glia, were selected for a Z-Max-Project performance. Then the cytoplasmic area of each neuron in the Z-MAX-Project image was circled to obtain the average value. For monitoring the fluorescent intensity of WT or A53T α-syn-mEos3.2 in neuron soma, the cytoplasmic area was circled after a Z-Max-Project performance to get the mean value. For ratiometric analysis of the puncta of mKeima and FIRE-pHLy as well as the immunofluorescence (IF) of LAMP1, the plugin Time series Analyzer 3.2 was used to set ROIs defined as 0.66 × 0.66 μm (six × six pixels) at the soma or axons. For analyzing the LAMP1-GFP puncta at the axons, the plugin KymoAnalyzer V1.01 (Neumann et al., 2017) was employed to measure the puncta density. For analyzing the fluorescent intensity of a-syn/a-syn A53T at axons, the tool Segmented Line [width: 0.33 μm (three pixels)] is used to obtain the average value through tracing the axons.

## Results

### *Synj1*+/− MB neurons exhibit normal autophagosome numbers and acidity at the basal level

**Figure 1.**
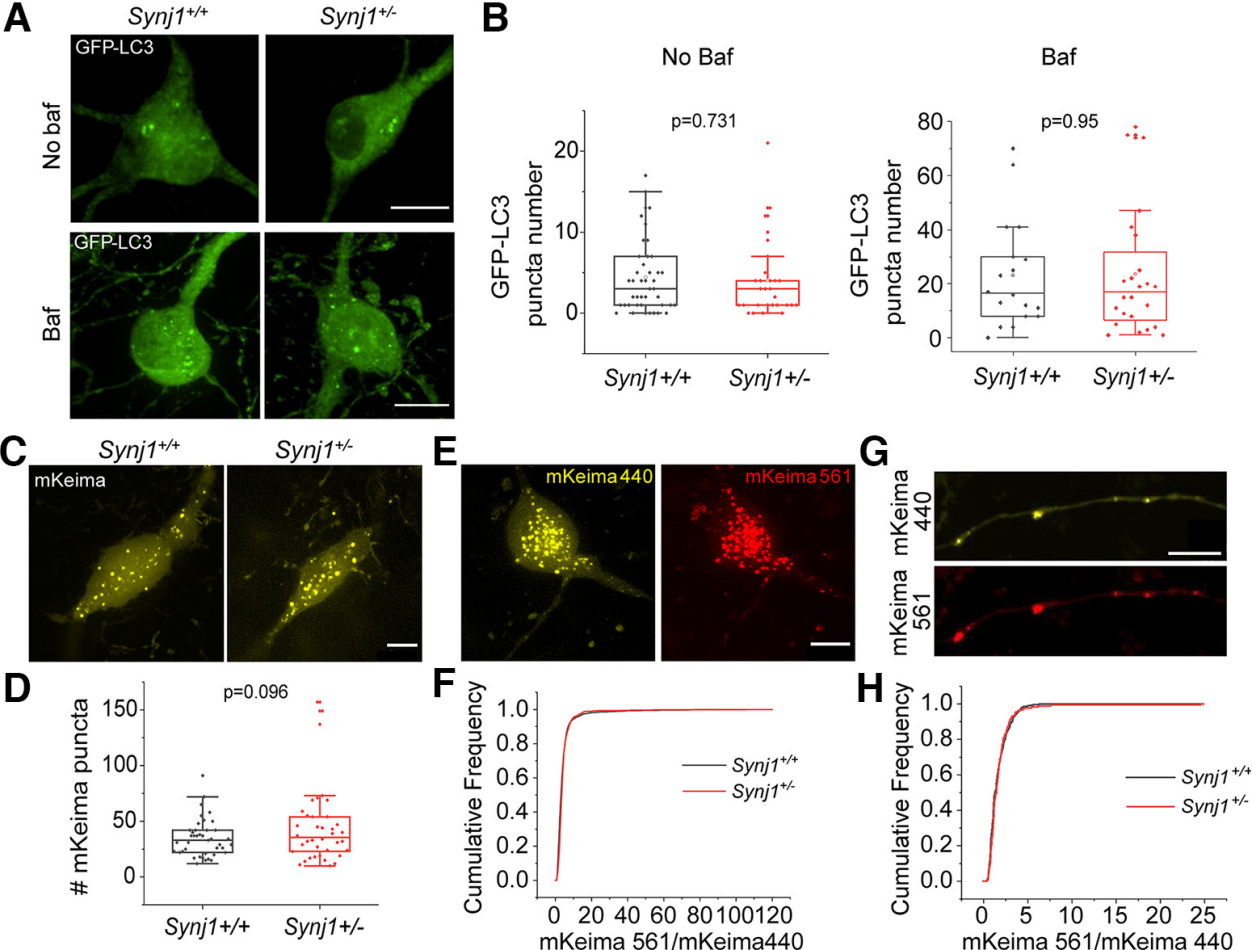
Synj1 deficiency does not substantially alter baseline autophagosome formation and flux in cultured MB neurons. ***A***, ***B***, GFP-LC3-labeled autophagosome in the soma of *Synj1+/+* and *Synj1*+/− MB neurons treated with/without 25 nm bafilomycin A1 for 6 h. ***A***, Representative images. ***B***, Quantification for the GFP-LC3 numbers at the soma. For no baf group, *N* = 44/29 (*Synj1+/+*/*Synj1*+/−) neurons, for baf group, *N* = 18/24 (*Synj1+/+*/*Synj1*+/−) neurons. ***C***, ***D***, mKeima-labeled autophagosome in the soma of *Synj1+/+* and *Synj1*+/− MB neurons. ***C***, Representative images. ***D***, Quantification for the mKeima puncta numbers at the soma, *N* = 41/38 (*Synj1+/+*/*Synj1*+/−) neurons. ***E***, Representative images show mKeima signal in the soma of MB neuron following activation by 440- and 561-nm light. ***F***, Distribution analysis of the ratio (mKeima561/mKeima440) for the mKeima puncta between *Synj1+/+* and *Synj1*+/− MB neurons, *N* = 2355/899 (*Synj1+/+*/*Synj1*+/−) puncta. ***G***, Representative images show mKeima signal in the axon of MB neuron from activation by 440 and 561 nm. ***H***, Distribution analysis of the ratio (mKeima561/mKeima440) for the mKeima puncta between *Synj1+/+* and *Synj1*+/− MB axons, *N* = 297/177 (*Synj1+/+*/*Synj1*+/−) puncta. The *p* values are from Student’s *t* test. Scale bar in all images: 10 μm.

Prior research has shown that in the brains of aged (12-month) male *Synj1*+/− mice, p62 is increased in the cortex, striatum, and MB DA neurons; however, enhanced LC3B level was only observed in the cortex and striatum but not in the MB DA neurons (Pan et al., 2020). To further investigate whether Synj1 regulates autophagy in MB neurons, we first transfected GFP-LC3 in cultured MB neurons from littermate *Synj1+/+* and +/− mice. All analyses were performed in mature neurons between days *in vitro* (DIV)13 and DIV16. Bafilomycin (Baf; 25 nm) was applied to the culture for 6 h to reveal the basal level autophagy flux. In both MB cultures treated with vehicle or baf, we found similar numbers of GFP-LC3 puncta in the cell bodies between *Synj1+/+* and *Synj1*+/− MB neurons (Fig. 1*A*,*B*), suggesting normal basal autophagy flux. GFP-LC3 puncta in the axons could not be determined with confidence in either *Synj1+/+* or *Synj1*+/− neurons and were therefore not included in the analysis. We next employed another molecular probe, mKeima, a pH-sensitive ratiometric fluorescent protein that is resistant to the lysosomal acidic environments. The mKeima is a cytosolic protein and mKeima puncta have been used as a measure of cumulative autophagic activity (Katayama et al., 2011). Less acidic (pH6–pH7) autophagosomes exhibit strong emission at 440-nm excitation but weak emission at 561-nm excitation, while more acidic autolysosomes (pH4–pH6) exhibit reverted emission ratio. No significant change in the mKeima puncta was observed in the *Synj1*+/− neuronal soma overtime (within 7 d from transfection to imaging) compared with the *Synj1+/+* neurons (Fig. 1*C*,*D*). Compared with GFP-LC3, mKeima was able to reveal a few more punctate structures along the axons (Fig. 1*G*); however, overall, these puncta were extremely sparse to justify an accurate analysis for axonal density. Thus, we only compared the acidity of mKeima structures by calculating the ratio of emissions at 440- and 561-nm excitations (Fig. 1*E–H*). We found that Synj1 deficiency did not change the acidity of mKeima structures either at the soma (Fig. 1*F*) or the axons (Fig. 1*G*,*H*) of MB neurons. Therefore, by expressing GFP-LC3 and mKeima in cultured MB neuron, we were unable to identify a significant contribution of Synj1 to autophagosome number and acidity at neuronal soma at the basal level.

### Synj1 deficiency is associated with changes in the lysosomal-associated membrane proteins (LAMPs) in cultured MB neurons

**Figure 2.**
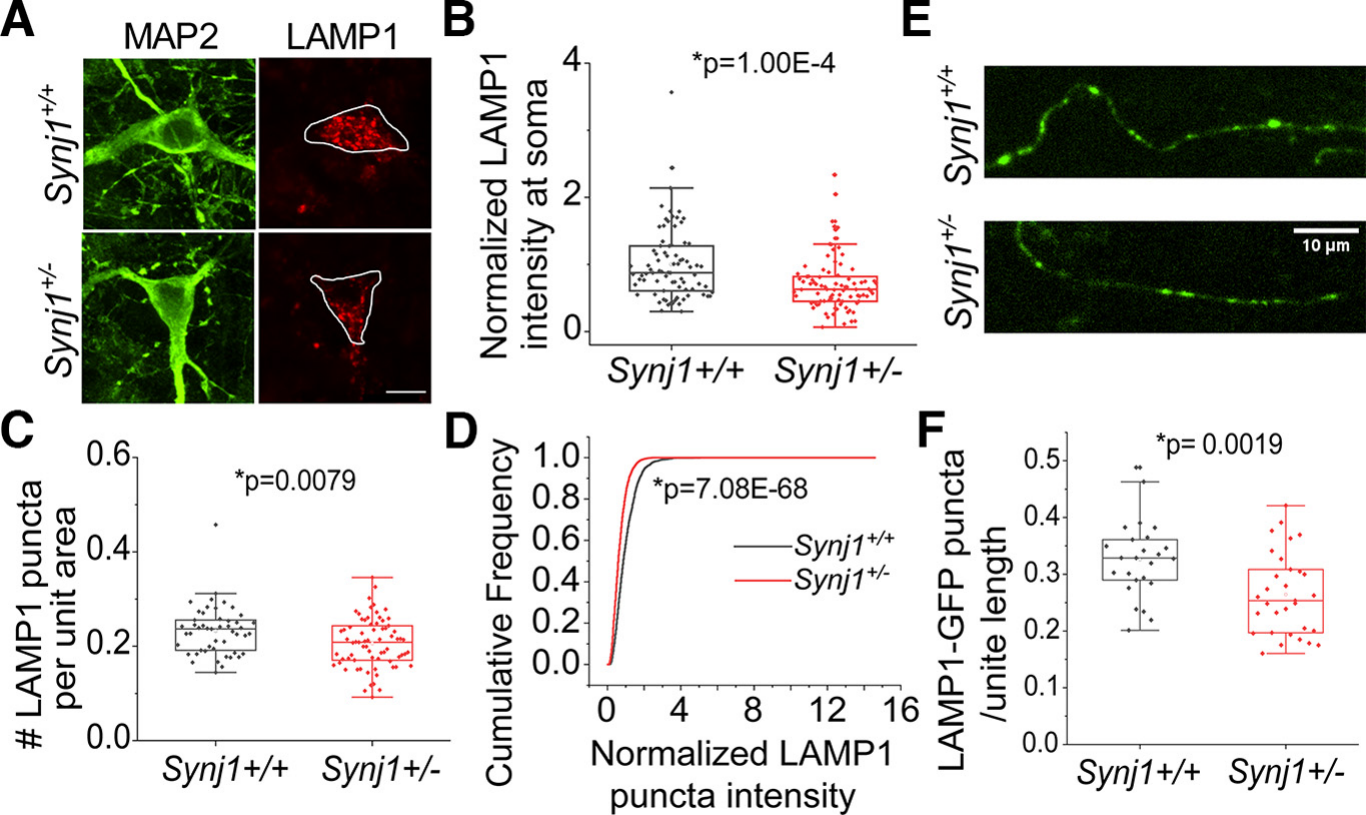
Reduced LAMP1 expression and puncta number in Synj1-deficient MB neurons. ***A***, ***B***, Immunofluorescence analysis of LAMP1 in the soma of *Synj1+/+* and *Synj1*+/− MB neurons, MAP2 is used as neuronal marker. ***A***, Representative images. ***B***, Quantification results, *N* = 82/88 (*Synj1+/+*/*Synj1*+/−) neurons. Extended Data Figure 2-1 shows reduced LAMP2 and LAMP2A in the soma of Synj1-deficient MB neurons. ***C***, Quantification of LAMP1-labeled puncta for *Synj1+/+* and *Synj1*+/− MB neurons. *y*-axis represents the normalized puncta number per unit cytoplasmic area, *N* = 82/88 (*Synj1+/+*/*Synj1*+/−) neurons. ***D***, Cumulative frequency graph shows the LAMP1 fluorescence intensity distribution of the puncta in soma between *Synj1+/+* and *Synj1*+/− MB neuron. *N* = 2091/2873 (*Synj1+/+*/*Synj1*+/−) puncta. ***E***, ***F***, Analysis of LAMP1-GFP puncta in the axons of *Synj1+/+* and *Synj1*+/− MB neurons. ***E***, Representative images. ***F***, Quantification results, *N* = 25/31 (*Synj1+/+*/*Synj1*+/−) axons. The *p* values for ***B***, ***C***, and ***F*** are from Student’s *t* test. The *p* value for ***D*** is from two-sample Kolmogorov–Smirnov test. Scale bar in all images: 10 μm. Extended Data Figure 2-2 shows the comparison of LAMP1/LAMP2A expression between TH+ and TH− MB neurons.

We next investigated the endolysosomal system by examining endogenous expression of lysosome-associated proteins, including LAMP1, LAMP2, and LAMP2A. We found that in the soma of *Synj1*+/− MB neurons the expression of LAMP1 was reduced by ∼25% (Fig. 2*A*,*B*). Additionally, the density of LAMP1 structures was significantly lower (Fig. 2*C*), accompanying a reduction in the immunofluorescence of each LAMP1 puncta in *Synj1*+/− MB neurons (Fig. 2*D*), suggesting a possible reduction of lysosome number and protein expression. Recent studies suggested that LAMP1 is not a reliable marker for lysosomes because of its presence on late endosomes (Cheng et al., 2018a, b). We thus performed immunofluorescence for two additional LAMP proteins: LAMP2 and LAMP2A. LAMP2A is a specific isoform of LAMP2 that regulates chaperone-mediated autophagy (CMA; Cuervo and Dice, 1996; Cuervo et al., 2004). Consistently, we found a near 25% reduction in both LAMP2 (Extended Data Fig. 2-1*A*,*B*) and LAMP2A (Extended Data Fig. 2-1*C*,*D*) at the cell bodies of *Synj1*+/− MB neurons. In both TH+ and TH− neurons from the midbrain, similar reductions of lysosomal associated proteins were observed (Extended Data Fig. 2-2). The differences among TH+ neurons were less significant likely because of a smaller sample size. Nevertheless, our results suggest Synj1 deficiency reduces the levels of LAMPs in ventral MB neurons. Because of the enriched expression of Synj1 in the axons and synapses, we next sought to examine whether Synj1 deficiency affects axonal lysosomal associated proteins. Using immunofluorescence, it was technically challenging to analyze these proteins at the axons in the mixed neuron-glia culture. We therefore expressed LAMP1-GFP in cultured MB neurons. Similarly, we found that the LAMP1 puncta density in the axons were significantly reduced in *Synj1*+/− MB neurons (Fig. 2*E*,*F*). Together, these results suggest a previously unknown role of Synj1 in regulating lysosomal associated proteins.

10.1523/ENEURO.0426-22.2023.f2-1 Extended Data Figure 2-1 Reduced LAMP2 and LAMP2A in Synj1-deficient MB neurons. ***A***, ***B***, Immunofluorescence analysis of LAMP2 in the soma of *Synj1+/+* and *Synj1*+/− MB neurons, MAP2 is used as neuronal marker. ***A***, Representative images. ***B***, Quantification results, *N* = 45/47 (*Synj1+/+*/*Synj1*+/−). ***C***, ***D***, Immunofluorescence analysis of LAMP2A in the soma of *Synj1+/+* and *Synj1*+/− MB neurons, MAP2 is used as neuron marker. ***C***, Representative images. ***D***, Quantification results, *N* = 158/144 (*Synj1+/+*/*Synj1*+/−). The *p* values for ***B*** and ***D*** are from Student’s *t* test. Scale bar in all images: 10 μm. Download Figure 2-1, TIF file.

10.1523/ENEURO.0426-22.2023.f2-2 Extended Data Figure 2-2 Comparison of LAMP1/LAMP2A expression between TH+ and TH− MB neurons. ***A***, ***B***, Box plots show the expression level of LAMP1 (***A***) and LAMP2A (***B***) between TH+ and TH− MB neurons for both *Synj1+/+* group and *Synj1*+/− group. For ***A***, *N* = 51/57/31/31 (TH− *Synj1+/+*; TH− *Synj1*+/−; TH+ *Synj1+/+*; TH+ *Synj1*+/−). For ***B***, *N* = 119/106/38/38 (TH− *Synj1+/+*; TH− *Synj1*+/−; TH+ *Synj1+/+*; TH+ *Synj1*+/−). The *p* values are from Tukey’s *post hoc* following Two-way ANOVA analysis. Download Figure 2-2, TIF file.

### Hyperacidification of LAMP1 vesicles and increased protease activity in Synj1-deficient MB neurons

**Figure 3.**
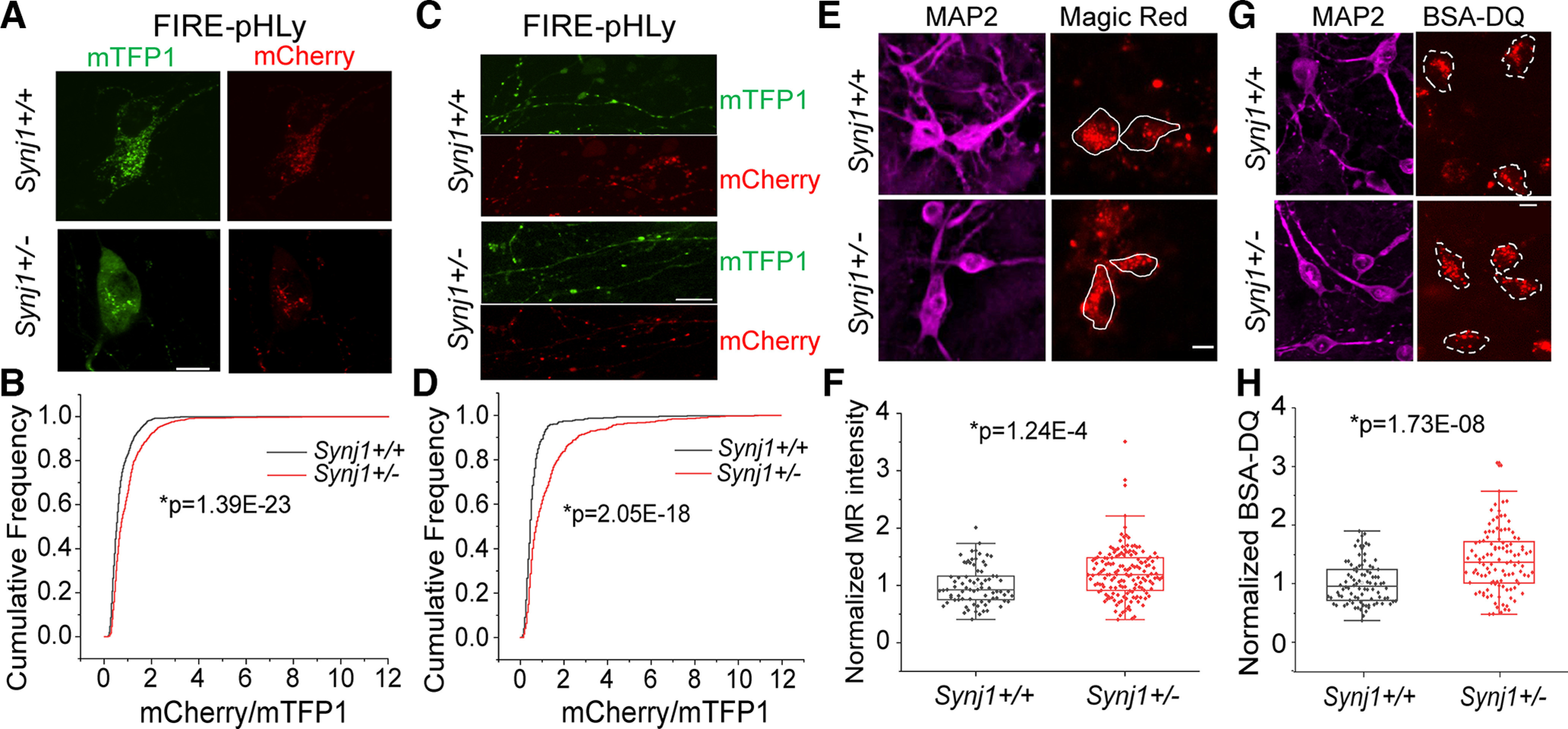
Synj1 deficiency leads to endolysosomal hyperacidification and enhanced enzymatic activity. ***A–D***, Endolysosomal acidity assay using a ratiometric fluorescent probe pHLy. ***A***, ***C***, representative images showing the mTFP1 signal and mCherry signal in the soma (***A***) and axons (***C***) of MB neurons transfected with pHLy. The brightness and contrast were adjusted to the same range for mTFP1 and mCherry channels. ***B***, ***D***, Distribution analysis of the ratio (mCherry/mTFP1) for the pHLy-labeled puncta between *Synj1+/+* and *Synj1*+/− MB neurons at the soma (***B***) and at the axons (***D***). *N* = 1151/1108 (*Synj1+/+*/*Synj1*+/−) puncta for soma analysis. *N* = 432/579 (*Synj1+/+*/*Synj1*+/−) puncta for axon analysis. ***E***, ***F***, Cathepsin-B activity analysis using the Magic Red dye. ***E***, Representative images showing the Magic Red signal from live cell imaging and the MAP2 signal from *post hoc* staining for *Synj1+/+* and *Synj1*+/− MB neurons. ***F***, Quantification of the intensity of Magic Red signal between *Synj1+/+* and *Synj1*+/− MB neurons. *N* = 78/159 (*Synj1+/+*/*Synj1*+/−) neurons. Extended Data Figure 3-1 shows that Synj1 deficiency does not alter the Cathepsin B expression in MB neurons. ***G***, ***H***, Analysis of endolysosomal protease activity using BSA-DQ. ***G***, Representative images. ***H***, Quantification results, *N* = 90/111 (*Synj1+/+*/*Synj1*+/−) neurons. The *p* values for ***B*** and ***D*** are from two-sample Kolmogorov–Smirnov test, and those for ***F*** and ***H*** are from Student’s *t* test. Scale bar in all images: 10 μm.

We next sought to determine whether function of the degradational vesicles, such as acidification and enzymatic activity, is impaired in Synj1-deficient MB neurons. To investigate acidity, we expressed a ratiometric lysosomal pH biosensor, Fluorescence Indicator REporting pH in Lysosomes (FIRE-pHLy, hereafter called pHLy; Chin et al., 2021). PHLy was engineered by tagging a cyan pH-sensitive fluorescent protein variant, mTFP1 (pK_a_ = 4.3), to the lumenal portion of the human LAMP1 cDNA and an mCherry to the cytosolic side. It was shown that the pHLy biosensor is targeted to express on LAMP1 vesicles and reports lumenal pH between 3.5 and 6 (Chin et al., 2021). Surprisingly, our live cell analysis found that endolysosomal acidity was increased in Synj1-deficient neurons both at the soma (Fig. 3*A*,*B*) and at the axon (Fig. 3*C*,*D*). The hyperacidified endolysosomes may result in elevated enzymatic activity in Synj1-deficient neurons. To test this, we performed two live cell staining assays to analyze cathepsin B activity and general protease activity. For cathepsin B analysis, we incubated the cultures with Magic Red, a substrate of the lysosomal cathepsin-B enzyme that fluoresces red on cleavage by active cathepsin enzymes. As expected, we found that the MR fluorescence was increased in *Synj1*+/− neurons (Fig. 3*E*,*F*) without changes in the cathepsin B expression level (Extended Data Fig. 3-1), indicating enhanced enzymatic activity. To further verify a general increase in endolysosomal protease activity, we next conducted a DQ-red BSA (DQ-BSA) assay. Like Magic Red, DQ-BSA is a fluorogenic substrate that fluoresces on hydrolysis by proteases. Consistently, we found increased DQ-BSA fluorescence in *Synj1*+/− neurons (Fig. 3*G*,*H*), suggesting an increase in overall protease activity.

10.1523/ENEURO.0426-22.2023.f3-1 Extended Data Figure 3-1 Synj1 deficiency does not alter the Cathepsin B expression in MB neurons. ***A***, Representative images showing the immunostaining signal of MAP2 and Cathepsin B in *Synj1+/+* and *Synj1*+/− MB neurons. ***B***, Fluorescent intensity quantification of Cathepsin B for the neurons in ***A***. *N* = 41/54 (Synj1+/+/Synj1+/−) neurons. The *p* value for ***B*** is from Student’s *t* test. Scale bar in ***A***: 10 μm. Download Figure 3-1, TIF file.

### Endolysosome defect is associated with Synj1’s SAC1 activity

**Figure 4.**
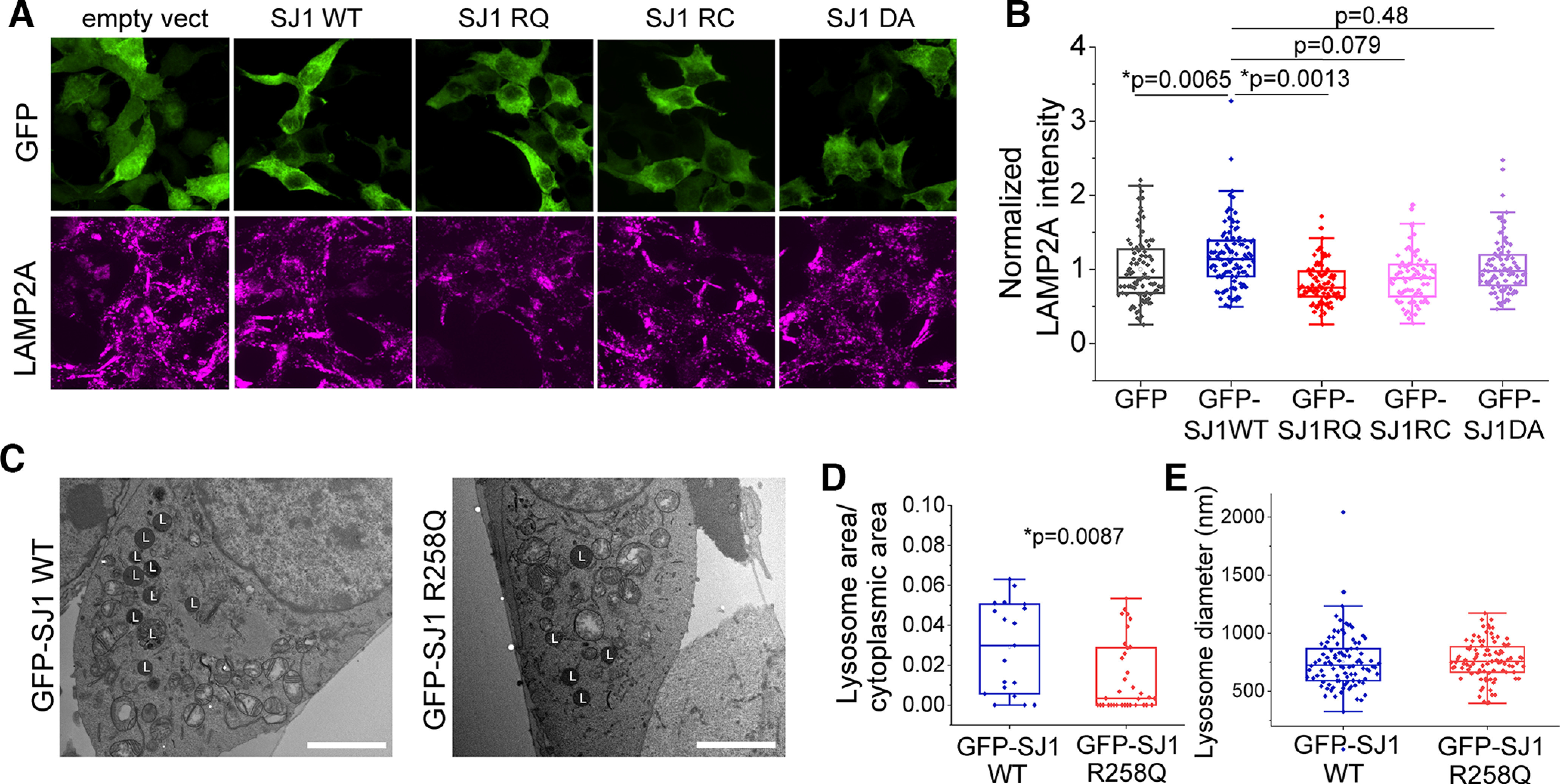
The SAC domain of Synj1 regulates endolysosomal abundance in N2a cells. ***A***, ***B***, Immunofluorescence analysis of LAMP2A in N2a cells transfected with GFP, GFP-hSYNJ1 WT, GFP-hSYNJ1 R258Q, GFP-hSYNJ1 R839C, and GFP-hSYNJ1 D769A. ***A***, Representative images. ***B***, Quantification results, *N* = 91/104/88/80/84 (GFP/GFP-SJ1WT/GFP-SJ1RQ/GFP-SJ1RC/GFP-SJ1DA) cells. *p* values are from Tukey’s *post hoc* following one-way ANOVA. ***C–E***, EM analysis of lysosome in N2a cells transfected with GFP-SJ1 WT and GFP-SJ1 R258Q. ***C***, Representative EM images. L, lysosome. ***D***, Quantification of the ratio of lysosome occupied area to cytoplasmic area in EM images for the two groups, *N* = 19/35 (*Synj1+/+/Synj1*+/−) ROIs. ***E***, Quantification of lysosome diameter in the EM images of the two groups, *N* = 97/95 (*Synj1+/+/Synj1*+/−) lysosomes. The *p* value is from Student’s *t* test. Scale bars: 10 μm (***A***) and 1 μm (***C***).

To further understand how the two enzymatic domains of Synj1 regulates endolysosomes, we expressed wild-type (WT) and mutant human SYNJ1 (SJ1) cDNAs in Neuro 2a (N2a) cells. Among all mutants we examined, SJ1 R258Q and SJ1 R839C were previously identified in Parkinsonism patients (Krebs et al., 2013; Quadri et al., 2013; Taghavi et al., 2018). The R258Q mutation abolishes SAC1 activity, and the R839C mutation reduces both SAC1 and 5’-phosphatase activities (Pan et al., 2020). The SJ1 D769A is not disease linked but abolishes 5’-phosphatase activity (Mani et al., 2007). Overexpressing WT SJ1 led to an increase in LAMP2A immunofluorescence compared with the empty GFP vector expressing cells (Fig. 4*A*,*B*). In contrast, the SJ1 R258Q mutant significantly downregulated LAMP2A expression, whereas the SJ1 R839C mutant produced a less significant effect on downregulating LAMP2A (Fig. 4*A*,*B*). Interestingly, the SJ1 D769A mutation did not alter the LAMP2A expression, suggesting that SAC1 rather than 5’-phosphatase activity is involved in LAMP2A regulation. We further performed electron microscopy (EM) analysis for SJ1 R258Q-expressing cells sorted by a fluorescence-activated cell sorting (FACS) system (Fig. 4*C*). Compared with those expressing WT SJ1, we found the lysosome density was decreased in N2a cells transfected with SJ1 R258Q (Fig. 4*D*), while the lysosome size was not affected (Fig. 4*E*). These results, taken together, suggested that the Synj1’s SAC1 domain is important for regulating lysosome quantity.

### Synj1 deficiency impairs α-syn A53T degradation in the axon

**Figure 5.**
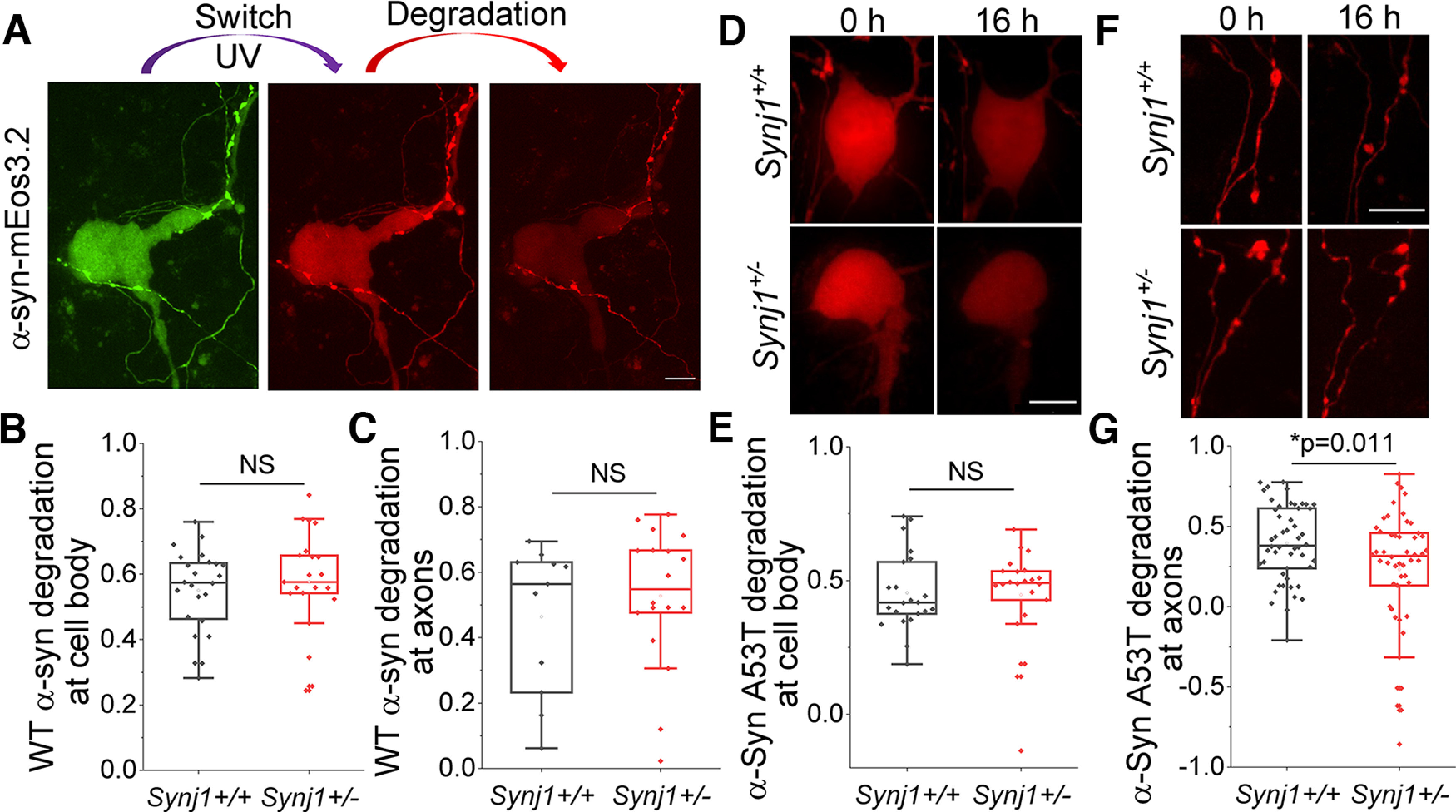
Synj1 deficiency impairs the degradation of α-Syn A53T in axons of cultured MB neurons. ***A–C*** , The α-syn WT-mEos3.2 degradation analysis at soma and axons in cultured MB neurons. ***A***, Representative images. ***B***, Quantification for the fraction of degradation of α-syn WT-mEos3.2 at soma within 16 hours. *N* = 24/21 (*Synj1+/+*/*Synj1*+/−) neurons. NS: *p* > 0.05 (two-tailed Student’s *t* test). ***C***, Quantification for the fraction of degradation of α-syn WT-mEos3.2 at axons, *N* = 11/18 (*Synj1+/+*/*Synj1*+/−) axons. NS: *p* > 0.05 (two-tailed Student’s *t* test). Extended Data Figure 5-1 shows that paraquat incubation induces similar impairments in the degradation of α-syn WT-mEos3.2 in *Synj1+/+* and *Synj1*+/− neurons. ***D–G***, The α-syn A53T-mEos3.2 16-hour degradation analysis at soma and axons in cultured MB neurons. ***D***, Representative images of soma. ***E***, Quantification for the fraction of degradation of α-syn A53T-mEos3.2 at soma. *N* = 22/22 (*Synj1+/+*/*Synj1*+/−) neurons. NS: *p* > 0.05 (two-tailed Student’s *t* test). ***F***, Representative images of axon. ***G***, Quantification for the fraction of degradation of α-syn-mEos3.2 A53T at axon, *N* = 50/50 (*Synj1+/+*/*Synj1*+/−) axons. The *p* value is from two-tailed Student’s *t* test. Scale bar in all images: 10 μm.

To determine the physiological impact of endolysosomal change in *Synj1*+/− neurons, we sought to investigate chaperone-mediated autophagy (CMA), which is mediated directly by LAMP2A. We hypothesized that the reduced LAMP2A would result in defective clearance of CMA substrates, such as the α-syn (Cuervo et al., 2004). We first assessed the degradation efficiency of the WT α-syn by tagging it to mEos3.2, a photo-switchable fluorescent protein. Upon UV irradiation, the green fluorescent α-syn-mEos3.2 will switch to a red-shifted protein (Zhang et al., 2012; Fig. 5*A*), allowing tracking of protein degradation without interference from nascent protein synthesis. In both cell bodies and axons of *Synj1*+/− neurons, we did not observe significant defects in α-syn-mEos3.2 protein degradation in a 16-hour chase experiment (Fig. 5*B*,*C*). More surprisingly, a 16-hour incubation of 2 μm paraquat, a widely studied PD-inducing herbicide and oxidant, was also not able to produce any defect in clearing WT α-syn in the *Synj1*+/− MB neurons (Extended Data Fig. 5-1), although a toxic effect on degradational capacity was observed comparing to naive conditions (Fig. 5*B*,*C*; Extended Data Fig. 5-1). We further investigated the degradation efficiency of α-syn A53T, a prevalent mutation of α-syn found in familial PD patients (Polymeropoulos et al., 1997). We found that cell bodies of *Synj1*+/− neurons could clear the UV converted α-syn A53T-mEos3.2 as effectively as the *Synj1+/+* neurons (Fig. 5*D*,*E*). However, the axons of *Synj1*+/− neurons exhibited a significant impairment (Fig. 5*F*,*G*), suggesting specific vulnerability in Synj1-deficient axons to protein stress. Taken together, our results suggest an impaired endolysosomal system in Synj1-deficient midbrain neurons, which could result in a specific vulnerability in axonal protein degradation.

10.1523/ENEURO.0426-22.2023.f5-1 Extended Data Figure 5-1 Paraquat incubation induces similar impairments in the degradation of WT-α-syn in *Synj1+/+* and *Synj1*+/− neurons. ***A–D***, The analysis of WT a-syn degradation at soma (***A***, ***B***) and axons (***C***, ***D***) of *Synj1+/+* and *Synj1*+/− MB neurons treated with Paraquat (PQ) for 16 hrs. ***A***, ***C***, Representative images for neuronal soma (***A***) and axons (***C***). ***B***, Quantification for the fraction of degradation of α-syn WT-mEos3.2 at soma. *N* = 30/17 (*Synj1+/+*/*Synj1*+/−) neurons. ***D***, Quantification for the fraction of degradation of α-syn WT-mEos3.2 at axons, *N* = 36/31 (*Synj1+/+*/*Synj1*+/−) axons. Scale bars in ***A***, ***C***: 10 μm. Download Figure 5-1, TIF file.

## Discussion

In this study, we performed a comprehensive analysis of cellular degradation systems in cultured *Synj1*+/− MB neurons. We found that baseline autophagy flux revealed by GFP-LC3 is largely unaltered in the neuronal cell body. However, we show, for the first time, that Synj1 regulates endolysosomal biology. The LAMP1 vesicle abundance is reduced in the soma and axons of *Synj1*+/− MB neurons, along with a general decrease in lysosomal-associated membrane proteins, including LAMP1, LAMP2, and LAMP2A. We show that the SAC1 activity of Synj1 is more important relative to the 5’-phosphatase activity for maintaining endolysosomal abundance. Interestingly, the acidity and enzymatic activity of the endolysosomal compartments are enhanced in *Synj1*+/− MB neurons, which may be a direct result of Synj1 deficiency or representative of compensatory changes. We further examined the impact of Synj1 deficiency on cellular degradation by chasing the fluorescence decrease of exogenously expressed α-syn WT and A53T mutant proteins. We found that the degradation of WT α-syn, which under normal conditions relies on LAMP2A-mediated CMA (Cuervo et al., 2004; Kuo et al., 2022) is normal in the cell body and axons of *Synj1*+/− MB neurons, suggesting adequate compensation. However, the A53T α-syn exhibited impaired degradation in the axons of *Synj1*+/− MB neurons, suggesting an axon-specific vulnerability in removing stress proteins.

A series of recent investigation suggested a regulatory role of Synj1 in various stages of autophagy. Contradictory to findings from the mouse astrocyte study that showed enhanced LC3 puncta in *Synj1*+/− and −/− backgrounds (Pan et al., 2021), and those from the *Drosophila* neuromuscular junction (NMJ) study that showed impaired LC3 puncta formation in the *Synj* RQ mutation knock-in background (Vanhauwaert et al., 2017), our study of the mouse *Synj1*+/− MB neurons suggested that Synj1 is not essential for maintaining basal level GFP-LC3 puncta and clearance at the neuronal cell body. This lack of change is likely because of lower metabolic demands at the neuronal soma at baseline compared astrocytes, and less membrane trafficking at the neuronal soma compared with an active synapse (i.e., NMJ). One limitation of the cell culture work is that it is difficult to reveal changes associated with age-related stress. A previous *in vivo* study in aged *Synj1* haploinsufficient (*Synj1*+/−) mice demonstrated an accumulation of the autophagy substrate p62 in the midbrain DA neuron cell bodies (Pan et al., 2020), suggesting that the unaltered LC3 clearance we observed in culture was likely because of compensation at a stress-free condition.

Synj1 has two phosphatase domains: the SAC1 domain enzyme that hydrolyzes PI(3)P, PI(4)P, and PI(3,5)P_2_; and the 5’-phosphatase domain enzyme that primarily hydrolyzes PI(4,5)P_2_. Different phosphoinositides are enriched in different subcellular compartments, acting as organizers of membrane organelles and participating in cellular signaling (Posor et al., 2022). For instance, studies have shown that despite the low abundance of PI(3,5)P_2_ in cells, it is implicated in autophagosome maturation (Vanhauwaert et al., 2017) and important for lysosome formation and ion homeostasis (McCartney et al., 2014; Hasegawa et al., 2017; Ebner et al., 2019). Synj1 deficiency could lead to an accumulation of PI(3,5)P_2_ to interfere with lysosomal function. In our study we showed that LAMP2A was decreased in *Synj1*+/− neurons and Synj1 R258Q-expresssing N2a cells, but not in Synj1 D769A or Synj1 R839C expressing cells. This suggests an involvement of SAC1 activity and, also, that the PI(3,5)P_2_ may negatively regulate endolysosomal protein expression. Indeed, PI(3,5)P_2_ is broadly involved in regulating lysosomal protein expression. It was found that PI(3,5)P_2_ can activate mTORC1 through direct binding to the mTORC1 subunit Raptor in yeast, which phosphorylates TFEB and, thereby, inhibits lysosomal gene expression (Bridges et al., 2012; Jin et al., 2014). Whether mTORC1 signaling is activated in *Synj1*+/− MB neurons to downregulate LAMP protein expression awaits further analysis.

Previous studies in yeast also shown that PI(3,5)P_2_ could directly bind to Vph1, a subunit of V-ATPase, to regulate V-ATPase assembly and proton pumping activity both *in vitro* and *in vivo* (Li et al., 2014; Banerjee et al., 2019). This is in line with our finding that endolysosomal acidity is significantly higher in *Synj1*+/− MB neurons compared with littermate WT neurons. Interestingly, while endolysosomes are much more acidic in *Synj1*+/− neurons than in *Synj1+/+* neurons, the acidity of the mKeima structures was not different. This could be attributed to the lack of power in the mKeima analysis, but it is also plausible that autophagosome-lysosome fusion is impaired in *Synj1*+/− MB neurons. For example, it was shown that PI(4,5)P_2_ not only mediates clathrin–AP2 coats assembly at the plasma membrane but also induces tubular protolysosome budding from tubular intermediates of autolysosomes (Rong et al., 2012), and participates in autophagosome and lysosome fusion (Posor et al., 2022). Whether the increased PI(4,5)P_2_ in *Synj1*+/− MB neurons (Pan et al., 2020) plays a role in disrupting the fusion between autophagosomes and lysosomes requires further investigation.

Despite finding multiple endolysosomal abnormalities in *Synj1*+/− MB neurons, we did not observe alterations in WT α-syn clearance at baseline or under paraquat stressed conditions. These data were striking as an accumulation of total and phosphorylated α-syn was reported in aged *Synj1*+/− male mice across multiple brain regions including the MB and striatum (Pan et al., 2020). Our data obtained from cultured MB neurons of mixed sex suggest several possibilities: (1) the degradation capacity of the MB neurons declines during aging, which is not captured in a culture system and is likely independent of oxidative stress; (2) the accumulation of α-syn proteins is a result of impaired glial function as opposed to neuronal degradation (Kam et al., 2020; Scheiblich et al., 2021); (3) the accumulation of α-syn proteins is a male-specific defect, which was diluted in the mixed sex culture. Interestingly, the degradation of the mutant protein, α-syn A53T, was significantly impaired in the axons of *Synj1*+/− neurons. Studies have shown that mutant α-syn exhibit impaired uptake by LAMP2A via the CMA pathway (Cuervo et al., 2004). Therefore, the impaired A53T a-syn degradation in *Synj1*+/− axons is likely because of mechanisms other than CMA, such as defective axonal autophagy, which was not extensively investigated in the current study because of technical limitations but supported by findings from invertebrate models of *Synj* deletion (Vanhauwaert et al., 2017; Yang et al., 2022). In summary, our data were largely consistent with findings from previous *in vivo* studies (Pan et al., 2020) and astrocyte cultures (Pan et al., 2021), which found that *Synj1* haploinsufficient neurons and glia become more vulnerable during aging with accumulating cellular stress. We now suggest that axons of *Synj1*+/− neurons are more vulnerable than cell bodies, supporting the dying back theory in Synj1 deficiency associated neurodegeneration (Bernheimer et al., 1973) and such axonal vulnerability could contribute in part to loss of dopaminergic innervation in aged Synj1-deficient brains (Pan et al., 2020).

## Synthesis

Reviewing Editor: Mikko Airavaara, Helsingin yliopisto Decisions are customarily a result of the Reviewing Editor and the peer reviewers coming together and discussing their recommendations until a consensus is reached. When revisions are invited, a fact-based synthesis statement explaining their decision and outlining what is needed to prepare a revision will be listed below. The following reviewer(s) agreed to reveal their identity: NONE.

The manuscript is very well written, experiments are well-performed, and result are well-organized. In summary, the findings can point towards a slight but significant slowness of degradation of a-synuclein A53T observed only in axons of SynJ1 haplo-insufficient cultured midbarin neurons compared to Synj1 +/+. This lagging degradation seems to be specific to the PD-associated a-syn A53T, since degradation of overexpressed WT a-syn is not affected. Finally, the study is building a hypothesis based on this idea that perhaps axonal compromised capacity to degrade proteins will initiate axonal injuries that ultimately cause neurodegeneration in a retrograde manner (dying back theory). Overall, the study is novel and very interesting for potential readers, however, the proposed mechanism is less robust to explain neurodegeneration in Synj1 +/- neurons. There are several concerns raised by the reviewers:

1. However, the data need to be carefully calibrated to the claims - and a great deal of care needs to be employed in using scientific terminology, which is not justified relative to the data presented. Simply reporting the data “as it is” and avoiding labels that have not been verified - would make for a more credible, precise and edifying manuscript. The comments are not intended to increase unnecessary experimental burden or workload - but merely to refine the text for clarity and precision. Please, revise the entire manuscript for typos, especially in the legends, such as “uM” or “nM” (for micro and nanometer) when authors clearly want to say “um” or “nm”. The central issue is the terminology used in the manuscript, which is inconsistent with the established conventions of the autophagy field. This inconsistency makes the article and resultant data challenging to interpret. The terms autophagosome, autolysosome and lysosome are used throughout the manuscript without experimental qualification, and they all mean very different things. For example, how do the authors distinguish between LAMP1-positive late endosomes and lysosomes? This distinction is not possible with the techniques presented. In addition, the central finding of lysosomal hyper-acidification is challenging to interpret because the authors have used lipofectamine - which directly modifies the autophagy pathway and intracellular lipid signaling (PMID: 20383065). Coupled with this, the expression of the lysosomal pH reporter is not endogenous - meaning it is challenging to dissociate expression artefacts from the basal state. The use of MR is welcomed, but the methods lack detail on how it is pulsed and quantified - intensity measurements are not ideal because segmentation is challenging, and these assays have a low dynamic range unless optimised extensively (optimisation data is lacking). These observations require verification with DQ-BSA.

2. The current iteration of the manuscript is not suitable for publication, and its novelty relative to prior literature is unclear and should be clarified (PMID: 34126070 - a study that uses many detailed approaches to label distinct cellular subcompartments).

3. Lysosomal diameter readings are derived from single optical sections and low-resolution images. However, these compartments are very dynamic and extend in the z-plane - so these measurements are also questionable. Terminal lysosomes are not acidic (!), a classical point of organelle biology often neglected in the literature (PMID: 27498570). Thus, it is unclear how to interpret the author’s experiments when aligned with gold-standard conventions and rigour in the field. Getting the simple details right would make for a much more coherent and convincing manuscript.

4. Another concern is that the authors used primary midbrain neurons; however, the reporter is incompatible with fixation. Thus, it is unclear whether the data are derived from reporter-expressing TH-positive neurons, which would represent a tiny percentage of the culture. The same concerns hold for the lysosomal pH reporter. Therefore, relevance to Parkinson’s disease should be made sparingly and with great care.

5. The authors repeatedly refer to mature autophagosomes, but there needs to be orthogonal data to verify that the structures quantified are, in fact, mature. These are assumptions because it is simply not possible using the approaches described - LC3 is one of several ATG8 proteins that decorates autophagy-related structures at different phases of the pathway, from nascent phagophore expansion to membrane scission. Many immunocytochemical analyses and TEM studies would be required to substantiate the stated claims of the authors in their current formulation. Indeed, ATG8s have many functions outside of degradative autophagy - and this has not been considered.

6. The authors could significantly improve the manuscript by introducing precision in the claims and text, i.e. the omission or exclusion of terms that are not directly qualified by experimental validation - and the introduction of a detailed limitations section that clearly outlines the caveats of the study. From the vantage point of this reviewer, the authors have investigated acidic endolysosomal compartments and LC3-positive structures, but this is not an autophagy study, and the analysis is limited in scope. There is nothing wrong with this; the authors should say what they did and what they observed relative to the constraints of the assays used - nothing more.

7. The reviewers appreciate that refining and streamlining the manuscript for precision and clarity might not be very exciting. But it is a better use of resources, time and intellectual capital that would yield an accurate and mature story that is, more importantly, likely to stand the test of time. This would also simplify the manuscript and expand its utility to the field.

8. If Synj1 deficiency in mouse neurons only affect degradation of a-syn A53T, how this mechanism can explain degeneration of DA-ergic neurons with no mutant a-synuclein. Is it possible that such a defect in protein degradation affect other axonal proteins that eventually cause axonal injury and neuronal degeneration? One possibility is that the axonal protein degradation capacity for a-syn WT is compromised but compensated through other mechanisms. To address this, it would be interesting to test the degradation of a-syn WT, while the system is challenged by other mechanisms such as oxidative stress. For example, would treatment with minimal doses of MPP+ or DA, show a difference in a-syn WT degradation between Synj1 +/+ and +/- axons?

9. Title adjustment: Synaptojanin1 modifies lysosomal parameters in cultured mouse neurons

10. “Autophagy (also known as, macroautophagy)”

“This process involves the formation of mature autophagosomes followed by fusion with lysosomes for degradation of cytosolic contents.”

This is incorrect. Autophagy is the general term for macroautophagy and selective autophagy, but it needs to be clarified what pathway the researchers are focusing on.

11. Referencing throughout the manuscript should follow a consistent style; line numbers would be helpful for the reviewers.

12. “the exact step where Synj1 is involved in the autolysosomal degradational pathway in mammalian neurons remains elusive.”

This study does not resolve this, so it should be omitted as a rationale.

13. “All analyses were performed in mature neurons between DIV (days in vitro) 13 and 16”

“Due to the lack of quenching, the number of autophagosomes observed at the cell body using mKeima (Fig. 1C-D) represents cumulative autophagosome formation at baseline, which was increased by ∼5-fold compared to those shown by GFP-LC3 (Fig. 1A-B).”

It is unclear whether all the structures are destined for degradation because of the autophagy-independent functions of LC3. This statement is not qualified in its current formulation. These experiments do not report autophagosome biogenesis - which would require far more mechanistic experimentation.

14. “We conclude that in cultured MB neuron, Synj1 does not contribute significantly to autophagosome formation and maturation at the basal level.”

This statement is not substantiated by the data presented.

15. Fig 1AB: Authors should compare No Baf vs Baf conditions too, to make sure their treatment is really increasing the number of LC3-positive structures.

16. Extended data Fig 2-2: Any suggestions why the TH- neurons show a decrease in LAMP1 and LAMP2A intensity but not in the TH+ depending on the Synj1 background? Is this relevant? Not mentioned in the results or discussion.

17. Fig 2E: Authors need to show a representative image for Synj+/- here.

18. Page 7, line 4: Authors should explain in one or two sentences how this reporter works so the reader doesn’t have to go to the reference or the mat & met to interpret the figure.

19. The last statement of the first paragraph of Page 7 (“indicating enhanced lysosomal enzymatic activity”) could also be explained by an increase in the amount of CathepsinB (or other cathepsins that degrade MR), so authors should check and compare the levels of (at least) CatB by WB to be able to claim this.

20. Fig 3CD: Please, show images of the two different backgrounds so the reader can understand how real are these differences. If not clear, I would suggest the authors provide a better biochemical result by immunoblotting the proteins of interest.

21. Page 9 authors write: “We found that the degradation of WT α-syn, which most likely relies on CMA is normal in the cell body and axons of Synj1+/- MB neurons.” However, authors show less LAMP2A and this protein is essential for CMA, so how do authors reconcile this statement with their results? And then, how can the authors explain the phenotype of the axonal deficiency in degradation of the mutant alpha-syn, when lysosomes move back and forth along the axon, potentially replenishing the axonal compartment from a soma that doesn’t show a deficient population of lysosomes?

22. Page 10: “This is in line with our finding that lysosomal acidity is significantly lower in Synj1+/- MB neurons”. I think authors meant ‘higher’ acidity. Please, revise.

23. Page 10: “...but it is also plausible that autophagosome-lysosome fusion is impaired in Synj1+/- MB neurons.” This statement is contradictory with their evidence: if that was the case, then authors should see a difference with mKeima that they don’t see. Please, explain.

24. Page 13, ‘Animals’ section: Can authors provide a number for their approved animal protocol? Also, some experimental details are missing (amount of plasmids transfected, for example?)

25. The study quality would be increased if the authors or other investigators have reported axonal degeneration of nigro-striatal neurons in a way that is specific to Synj1 haplodeficient mice (in vivo), at least this should be discussed
