# Supplementary figures and images for: Synaptojanin1 Modifies Endolysosomal Parameters in Cultured Ventral Midbrain Neurons
Source: eNeuro. 2023 May 4;10(5):ENEURO.0426-22.2023. doi: 10.1523/ENEURO.0426-22.2023 (PMC10166127; doi:10.1523/ENEURO.0426-22.2023)

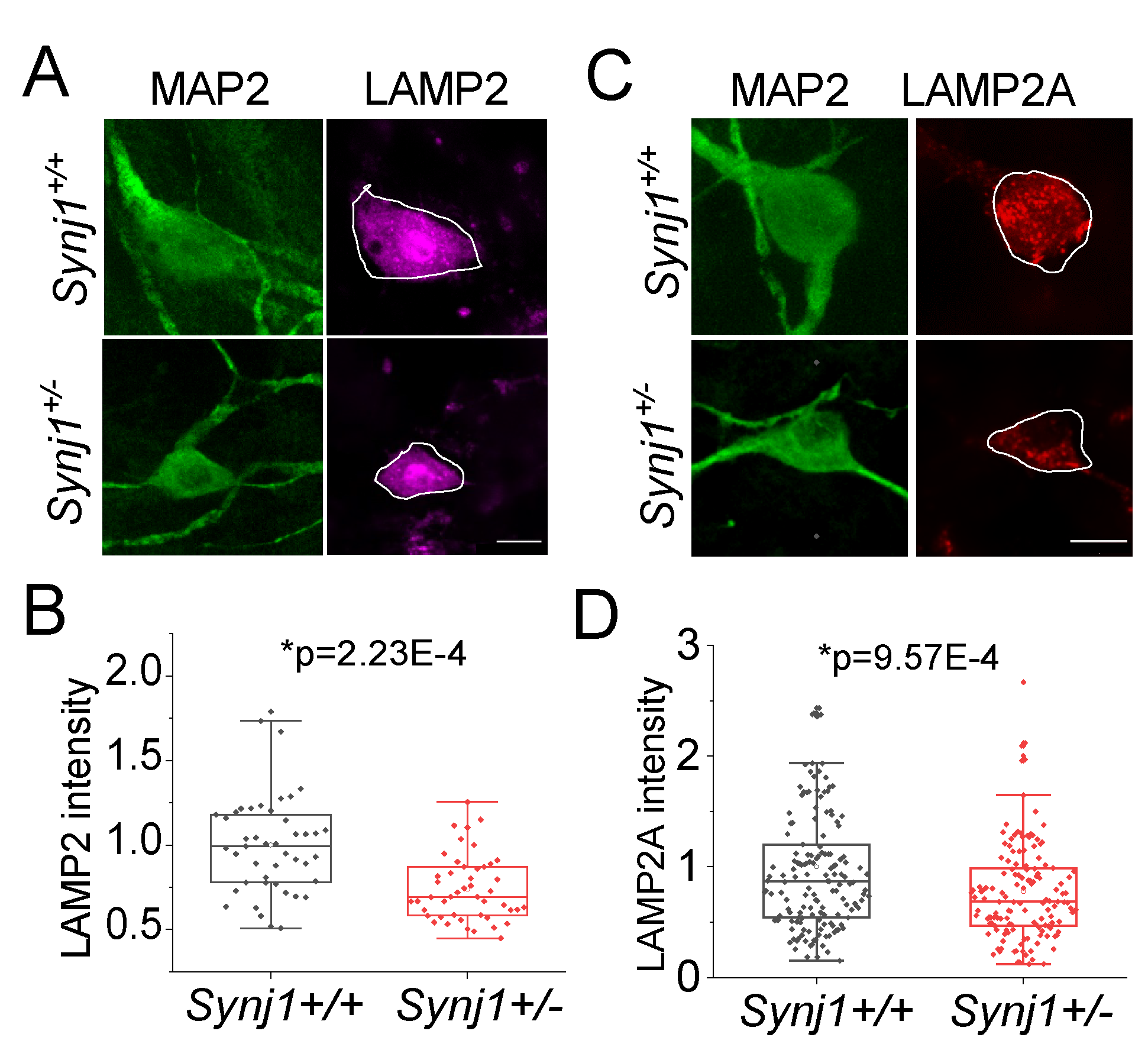

Supplement: Extended Data Figure 2-1 — Reduced LAMP2 and LAMP2A in Synj1-deficient MB neurons. A, B, Immunofluorescence analysis of LAMP2 in the soma of Synj1+/+ and Synj1+/− MB neurons, MAP2 is used as neuronal marker. A, Representative images. B, Quantification results, N = 45/47 (Synj1+/+/Synj1+/−). C, D, Immunofluorescence analysis of LAMP2A in the soma of Synj1+/+ and Synj1+/− MB neurons, MAP2 is used as neuron marker. C, Representative images. D, Quantification results, N = 158/144 (Synj1+/+/Synj1+/−). The p values for B and D are from Student’s t test. Scale bar in all images: 10 μm. Download Figure 2-1, TIF file. [file enu-eN-NWR-0426-22-s01.tif]

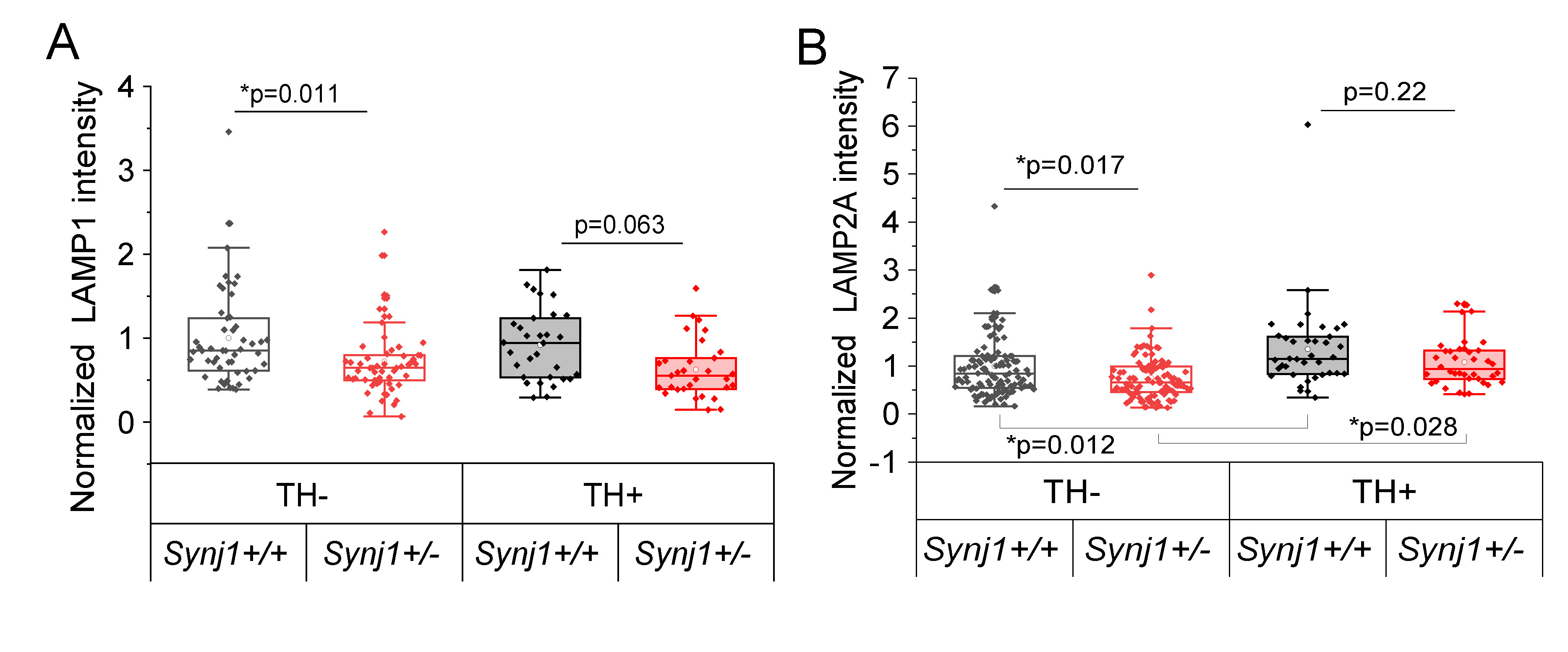

Supplement: Extended Data Figure 2-2 — Comparison of LAMP1/LAMP2A expression between TH+ and TH− MB neurons. A, B, Box plots show the expression level of LAMP1 (A) and LAMP2A (B) between TH+ and TH− MB neurons for both Synj1+/+ group and Synj1+/− group. For A, N = 51/57/31/31 (TH− Synj1+/+; TH− Synj1+/−; TH+ Synj1+/+; TH+ Synj1+/−). For B, N = 119/106/38/38 (TH− Synj1+/+; TH− Synj1+/−; TH+ Synj1+/+; TH+ Synj1+/−). The p values are from Tukey’s post hoc following Two-way ANOVA analysis. Download Figure 2-2, TIF file. [file enu-eN-NWR-0426-22-s02.tif]

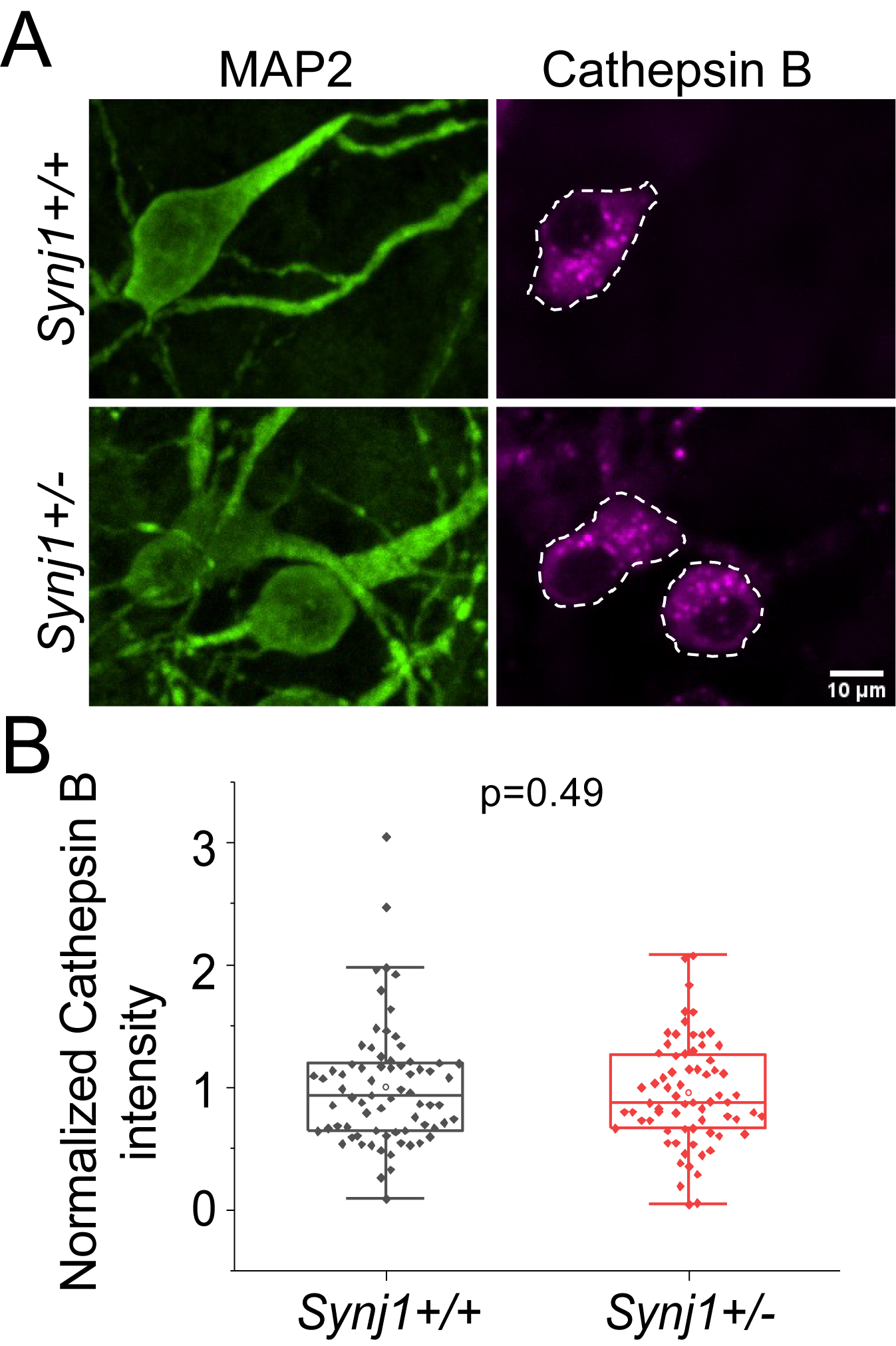

Supplement: Extended Data Figure 3-1 — Synj1 deficiency does not alter the Cathepsin B expression in MB neurons. A, Representative images showing the immunostaining signal of MAP2 and Cathepsin B in Synj1+/+ and Synj1+/− MB neurons. B, Fluorescent intensity quantification of Cathepsin B for the neurons in A. N = 41/54 (Synj1+/+/Synj1+/−) neurons. The p value for B is from Student’s t test. Scale bar in A: 10 μm. Download Figure 3-1, TIF file. [file enu-eN-NWR-0426-22-s03.tif]

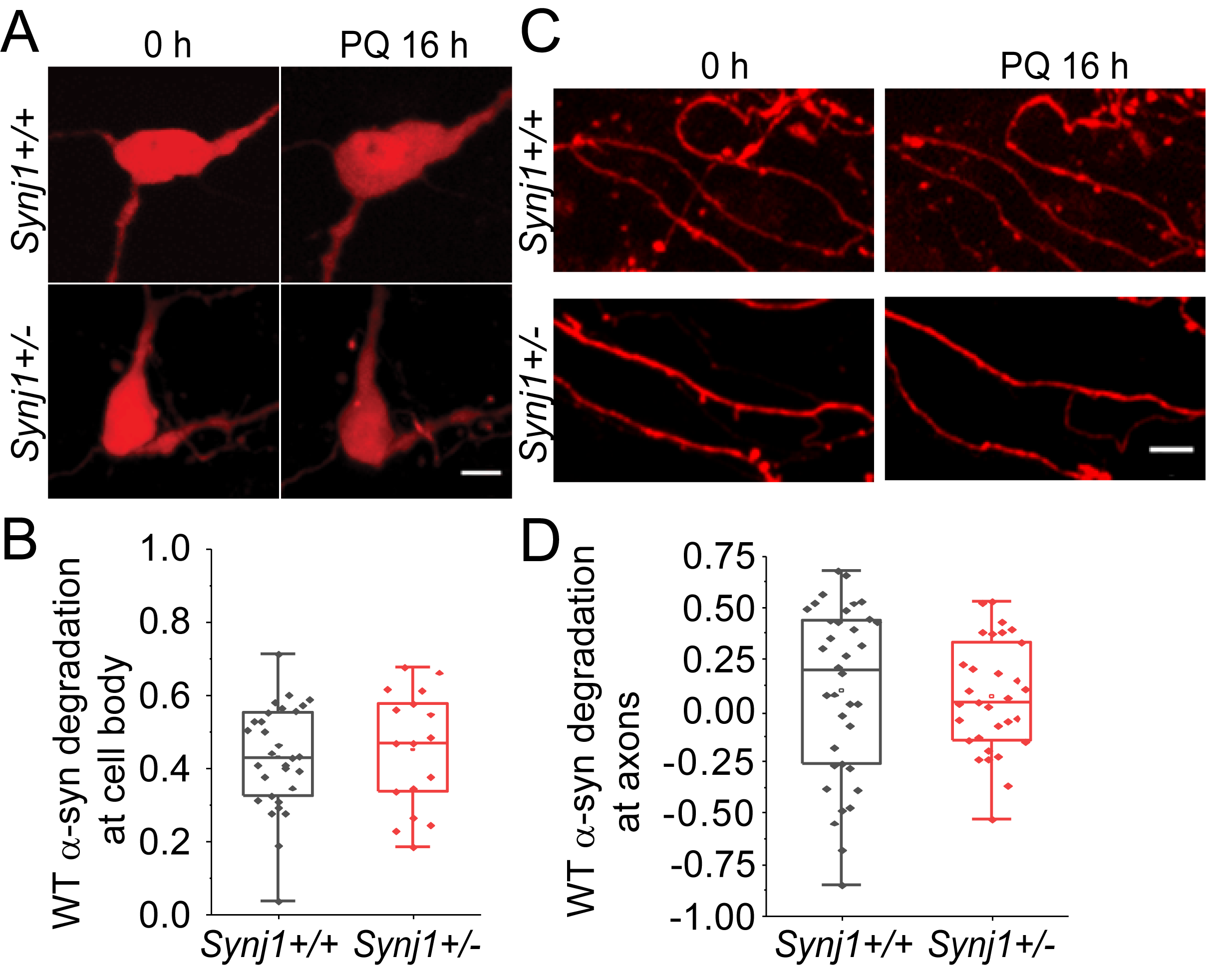

Supplement: Extended Data Figure 5-1 — Paraquat incubation induces similar impairments in the degradation of WT-α-syn in Synj1+/+ and Synj1+/− neurons. A–D, The analysis of WT a-syn degradation at soma (A, B) and axons (C, D) of Synj1+/+ and Synj1+/− MB neurons treated with Paraquat (PQ) for 16 hrs. A, C, Representative images for neuronal soma (A) and axons (C). B, Quantification for the fraction of degradation of α-syn WT-mEos3.2 at soma. N = 30/17 (Synj1+/+/Synj1+/−) neurons. D, Quantification for the fraction of degradation of α-syn WT-mEos3.2 at axons, N = 36/31 (Synj1+/+/Synj1+/−) axons. Scale bars in A, C: 10 μm. Download Figure 5-1, TIF file. [file enu-eN-NWR-0426-22-s04.tif]
